# Implementing pharmacogenetic testing in community pharmacy practice: a scoping review

**DOI:** 10.3389/fphar.2025.1659875

**Published:** 2025-09-22

**Authors:** Aude Coumau, Claire Coumau, Chantal Csajka

**Affiliations:** ^1^ Center for Research and Innovation in Clinical Pharmaceutical Sciences, Lausanne University Hospital and University of Lausanne, Lausanne, Switzerland; ^2^ Institute of Pharmaceutical Sciences of Western Switzerland, University of Geneva, Geneva, Switzerland; ^3^ Institute of Pharmaceutical Sciences of Western Switzerland, University of Lausanne, Geneva, Switzerland; ^4^ School of Pharmaceutical Sciences, University of Geneva, Geneva, Switzerland

**Keywords:** pharmacogenetics, scoping review, implementation science, community pharmacy services, precision medicine

## Abstract

The field of pharmacogenetics (PGx) has expanded significantly in recent years, with growing evidence supporting its role in enhancing medication effectiveness and reducing adverse drug events. Yet, the integration of PGx into routine clinical practice remains limited. Community pharmacies hold a key position in the healthcare system, offering expert medication advice and maintaining close patient contact due to their accessibility. This context has driven research efforts to integrate PGx testing into healthcare systems in various countries. However, evidence on optimal strategies for embedding PGx services in community pharmacy settings is still emerging. We conducted a scoping review to provide a comprehensive overview of the implementation of PGx testing in community pharmacies, focusing on both successful strategies and challenges. A systematic search of studies involving PGx testing in community pharmacies was conducted using PubMed, Embase, the Cochrane Library and Web of Science, including all publications up to February 2025. The search considered implementation outcomes: feasibility, acceptability, adoption, fidelity, appropriateness, cost, penetration and sustainability. The process and reporting followed the PRISMA recommendations for scoping reviews (PRISMA-ScR). Study findings were classified according to Proctor’s implementation outcomes. A total of 17 studies met the inclusion criteria and were included in the review. Key implementation variables were extracted from these studies. Feasibility was supported by a manageable time process and high technical success. The appropriateness of PGx was reflected in its ability to identify numerous medication-related issues. Adoption varied between patients and prescribers. While patient engagement was high, many sharing PGx results with other physicians, integration of PGx recommendations by prescribers was inconsistent. The intervention was generally well accepted, with high satisfaction among patients and pharmacists, although some physicians expressed concerns. These findings illustrate potential approaches to implementing PGx testing in community pharmacy settings. This scoping review demonstrates the potential for PGx testing to become a viable part of routine care in community pharmacies. It highlights positive patient perceptions and provider willingness to adopt testing. However, it also identifies key barriers, including the need for standardized PGx guidelines, education for providers, and reimbursement policies. The study underscores the importance of patient education, seamless integration into pharmacy workflows, and continued research to support successful implementation.

## 1 Introduction

The paradigm of pharmacotherapy has changed significantly in recent decades, transitioning from a “one-size-fits-all” treatment strategy to a more precise and individualized approach, commonly referred to as “personalized medicine”. This evolution is largely motivated by the urgent need to reduce adverse drug reactions (ADRs), which affect approximately 20% of patients in primary care (Insani et al., 2021), with a significant proportion considered preventable. Pharmacogenetics (PGx) has emerged as a key tool in this context, with PGx-guided prescribing shown to reduce clinically relevant ADRs by up to 30% (Swen et al., 2023). Beyond reducing ADRs, PGx-guided prescribing has also been shown to improve therapeutic effectiveness (Tesfamicael et al., 2024). The clinical relevance of PGx is further underscored by the development of companion diagnostics. For instance, HLA-B*57:01 genotyping is required prior to prescribing abacavir, as carriers are at high risk of severe hypersensitivity reactions (Martin et al., 2012). Similarly, *CYP2C19* genotyping is increasingly used to guide antiplatelet therapy with clopidogrel, identifying patients at risk of reduced response due to limited drug activation (Lee et al., 2022). Today, health technologies are increasingly being used in both clinical practice and research to guide mutation-targeted therapies. Indeed, numerous studies have demonstrated that PGx testing can optimize drug selection and dosing by identifying treatments more likely to be effective and better tolerated according to a patient’s genetic profile (Bollinger et al., 2023; Wick et al., 2003; Goetz and Schork, 2018). This assertion is supported by numerous studies showing that genetic variations affecting drug-metabolizing enzymes, transport proteins, receptors, or other pharmacological targets can significantly alter patient responses to common medications. For example, clinical response to selective serotonin reuptake inhibitors (SSRIs) is influenced by receptor and transporter polymorphisms such as those in the serotonin transporter gene *(SLC6A4)* (Stein et al., 2021), but also by variations in drug-metabolizing enzymes, particularly CYP2C19 and CYP2D6, which modulate plasma concentrations and ultimately affect treatment effectiveness and safety (Arnone et al., 2023). In oncology, *DPYD* variants can lead to severe toxicity with fluoropyrimidines (Amstutz et al., 2018), while *UGT1A1* polymorphisms are associated with irinotecan-induced neutropenia (Hulshof et al., 2020). Additionally, the HLA-B*58:01 allele strongly predicts the risk of life-threatening cutaneous reactions to allopurinol (Saito et al., 2016). Overall, genetic factors are estimated to account for approximately 20%–30% of the observed variability in drug response (Heller, 2013).

PGx information is now included in an increasing number of drug monographs, with approximately 360 published by the U.S. Food and Drug Administration (FDA) (Food and Drug Administration FDA, 2024). Similarly, the European Medicines Agency (EMA) has issued guidance on the integration of PGx testing into the drug approval process (European Medicines Agendy, 2024). However, despite this regulatory recognition, there remains a substantial gap in implementation in clinical practice. This discrepancy is largely explained by persistent barriers such as limited healthcare professional training in pharmacogenetic, restricted access to reliable and rapid testing, uncertainties regarding reimbursement and costs, and logistical challenges related to the integration of PGx into existing clinical workflows. In addition, several upstream factors contribute to this gap, including the relative novelty of the field, the fact that PGx represents only one of multiple sources of variability in drug response, and the limited but growing body of evidence on its clinical effectiveness and safety. Understanding these barriers is therefore critical to guide strategies that can bridge the gap between regulatory recommendations and real-world clinical adoption.

Among the healthcare professionals involved, community pharmacists may play a pivotal role in bridging this gap. In fact, community pharmacists are well positioned to deliver PGx testing services due to their easy accessibility (Valliant et al., 2022; Berenbrok et al., 2020), their role in medication revision and patient education (Breault et al., 2017) and their expanding clinical responsibilities in drug delivery, adjustment and monitoring (Berenbrok et al., 2022). This role has therefore been formally endorsed by several professional pharmacy organizations (Haidar et al., 2015; Owen, 2011; Smith et al., 2025). However, although evidence increasingly supports the use of PGx testing to enhance patient outcomes (Hayashi et al., 2022; Rendell et al., 2021; Bousman et al., 2017), its implementation in community pharmacy settings remains limited, highlighting a persistent gap between recommended and actual practice (Hayashi et al., 2022).

In this context, this scoping review aims to evaluate the current integration of PGx testing in community pharmacies and identify associated barriers and facilitators to support its implementation.

## 2 Materials and methods

### 2.1 Search strategy and selection criteria

This scoping review has been designed in accordance with the Preferred Reporting Items for Systematic Reviews and Meta-Analyses guidelines extension for Scoping Review (PRISMA-ScR) (Tricco et al., 2018). Systematic literature searches were conducted in PubMed, Embase, Web of Science and Cochrane Central Register of Controlled Trials (Wiley) on 25 March 2021, with an update on 5 February 2025, and with the assistance of a biomedical librarian. The search terms were the following: ‘community pharmacy’, ‘pharmacies’, ‘primary care’, ‘pharmacogenetics’, ‘pharmacogenomics’, ‘genetic testing’, ‘genetic counselling’, and ‘genetic sequencing’. Filters to exclude animal studies and conference abstracts were applied to the strategies, but no date or language limits were applied. The strategies were peer-reviewed according to the Peer Review of Electronic Search Strategies (PRESS) checklist (McGowan et al., 2016) by another librarian and are presented in Supplementary Material 1. References were imported into EndNote 20 (Clarivate™, London, UK) and deduplicated.

The inclusion criteria were the following (Insani et al., 2021): studies focused on the implementation of PGx tests within community pharmacies (Swen et al., 2023); population of physicians, pharmacists and patients (Tesfamicael et al., 2024); interventions involving PGx testing (Martin et al., 2012); outcomes comprising Proctors’ implementation variables (Proctor et al., 2011), including feasibility, acceptability, adoption, appropriateness, fidelity, implementation cost, penetration, and sustainability. This implementation variables were defined as themes and were further divided into sub-themes based on the data available in the included articles (see Supplementary Material 2). PGx tests conducted outside community pharmacy settings, used for non-prescription purposes, articles that were not original research or unavailable in full text were excluded.

This protocol is preregistered on the Open Science Framework (OSF) and is publicly accessible at the following link: https://doi.org/10.17605/OSF.IO/K5X7W.

No formal quality appraisal of included studies was performed, since the objective was to map the existing evidence rather than to assess the methodological quality of individual sources, as recommended in scoping review methodology.

### 2.2 Selection process

One investigator (ACO) screened titles and abstracts using Rayyan^®^ (rayyan.qcri.org) (Ouzzani et al., 2016), to facilitate this process. Subsequently, two investigators (ACO and CCO) independently performed the study selection process based on the full text of the articles for eligibility. Any disagreements regarding the study inclusion were resolved through discussions until a consensus was reached.

## 3 Results

### 3.1 Study selection

**FIGURE 1:**
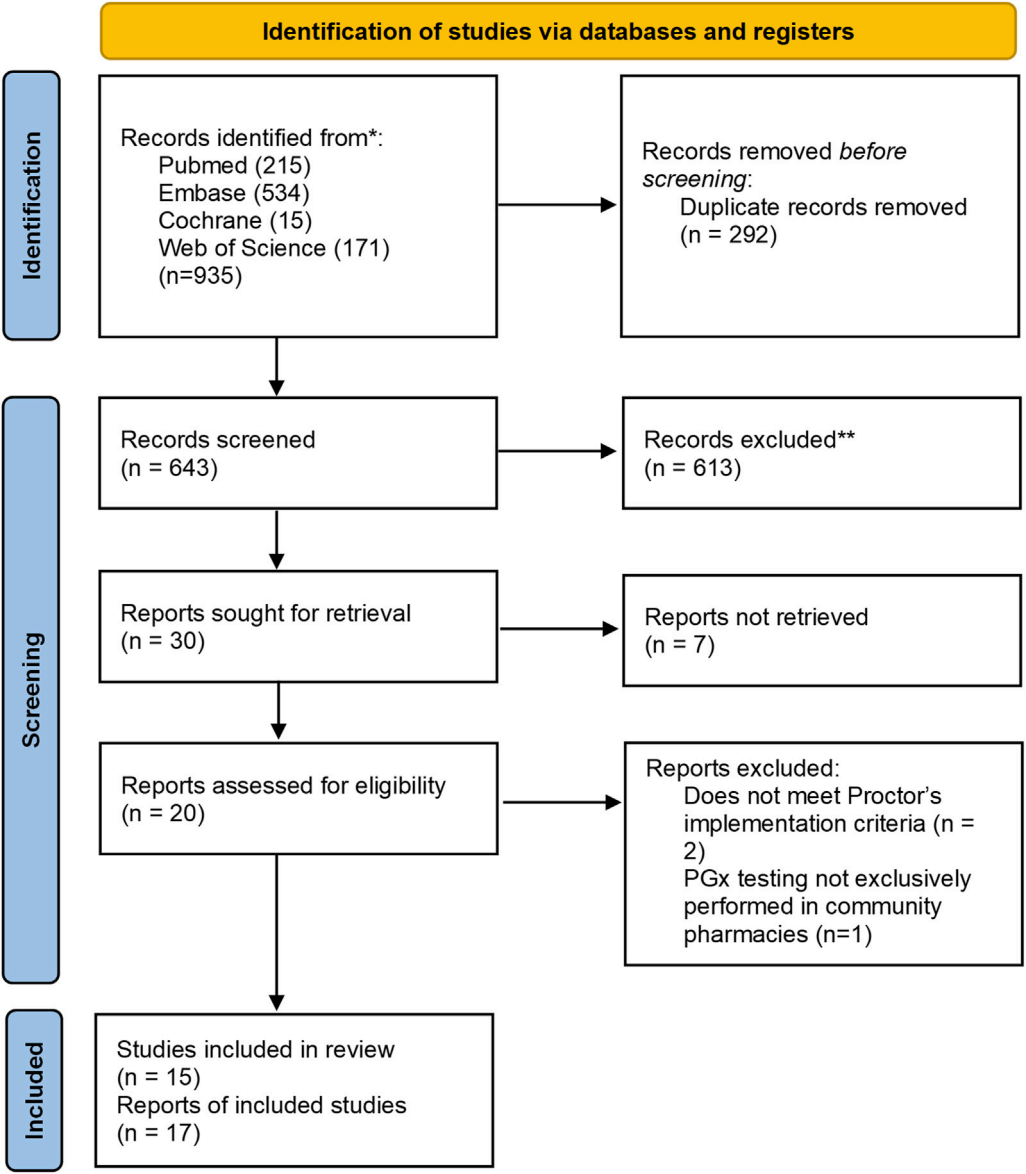
Preferred Reporting Items for Systematic Reviews and Meta-Analysis (PRISMA) diagram for the inclusion of studies assessing implementation of pharmacogenetic tests into community pharmacies.

We identified 935 articles with 643 remaining after deduplication. 613 irrelevant records were excluded based on the screening of titles and abstracts. We were unable to find 7 full-text reports and reviewed 20 reports in detail. Among these, 2 were excluded due to non-compliance with the Proctor implementation criteria and 1 was excluded due to the lack of exclusive PGx testing conducted in community pharmacies. Finally, 17 studies were included. The PRISMA diagram presenting the inclusion process is presented in Figure 1.

### 3.2 Study characteristics

Overall, 120 community pharmacies participated across the included studies (median [range]: 4 [1–27]), although 4 articles did not specify the number of pharmacies involved. A total of 124 pharmacists were included (median [range]: 14 [1–36]), with this information missing in 8 articles. Three articles documented the participation of physicians, totaling 237 individuals (median [range]: 21 [8–208]). Regarding patients, a total of 2,134 were included (median [range]: 109 [18–611]), with only 1 article failing to report this information. Study duration ranged from 3 to 32 months (median [range]: 11.5 [3–32]) among the 14 studies that specified this information. Six (35.2%) studies were carried out in Netherlands, 5 (29.4%) in the USA, 4 (23.5%) in Canada, 1 (5.8%) in Spain and 1 (5.8%) in Switzerland. Of the 17 included studies, 12 (70.6%) were quantitative (Bank et al., 2019; Breaux et al., 2020; Bright et al., 2015; Ferreri et al., 2014; Haga et al., 2021a; Levens et al., 2023; Moaddeb et al., 2015; Papastergiou et al., 2017; Papastergiou et al., 2021; Rodríguez-Arcas et al., 2013; Swen et al., 2012; Van Der Wouden et al., 2019), 1 (5.8%) was qualitative (Luke et al., 2021) and 4 (23.5%) were mixed methods, combining both qualitative and quantitative data (Haga et al., 2021b; Jeiziner et al., 2023; Thornley et al., 2021; Van Der Wouden et al., 2020). All studies were prospective in design: 6 (35.2%) were interventional (Bright et al., 2015; Haga et al., 2021a; Levens et al., 2023; Papastergiou et al., 2021; Rodríguez-Arcas et al., 2013; Haga et al., 2021b), including 3 randomized controlled studies (Haga et al., 2021a; Papastergiou et al., 2017; Haga et al., 2021b); among these, 2 were cluster randomized studies involving pharmacies (Haga et al., 2021a; Haga et al., 2021b). The remaining 11 studies (64.7%) were observational (Bank et al., 2019; Breaux et al., 2020; Ferreri et al., 2014; Moaddeb et al., 2015; Papastergiou et al., 2017; Swen et al., 2012; Van Der Wouden et al., 2019; Luke et al., 2021; Jeiziner et al., 2023; Thornley et al., 2021; Van Der Wouden et al., 2020). PGx training for pharmacists, either prior to or specifically for the study, was reported in most studies (94.1%), with only one exception (Swen et al., 2012). In contrast, none of the included studies reported any specific PGx training for physicians. Regarding the type of PGx testing performed, 7 studies (41.2%) used preemptive testing (Bank et al., 2019; Papastergiou et al., 2017; Rodríguez-Arcas et al., 2013; Swen et al., 2012; Luke et al., 2021; Thornley et al., 2021; Van Der Wouden et al., 2020), 9 (52.9%) used reactive testing (Breaux et al., 2020; Bright et al., 2015; Ferreri et al., 2014; Haga et al., 2021a; Levens et al., 2023; Moaddeb et al., 2015; Papastergiou et al., 2021; Haga et al., 2021b; Jeiziner et al., 2023), and 1 (5.8%) study was classified as hybrid: the test was performed reactively at the time of the first prescription and the results were subsequently stored for preemptive use in future prescribing decisions (Van Der Wouden et al., 2019). The median number [range] of pharmacogenes tested across studies was 6.5 [1–30], with 1 study not reporting this information (Luke et al., 2021). Saliva was used as the biological sample in all included studies. The characteristics of the included articles are presented in Table 1.

**TABLE 1:** Summary of characteristics of selected studies from the scoping review.

References	Country	Study population	Study design	Aims	Study duration
Bank et al. (2019)	Netherlands	200 patients	Prospective observational study	Investigate the adoption of pharmacist initiated PGx testing in primary care	32 months
Breaux et al. (2020)	Canada	180 patients, 21 pharmacists, 17 community pharmacies	Prospective observational study	Test feasibility and appropriateness of community pharmacies to propose PGx* testing Gauge the receptivity of patients in this setting Assess the cost-effectiveness of this approach	6 months
Bright et al. (2015)	USA	30 patients, 4 community pharmacies	Prospective multisite proof-of-concept interventional study	Present a proof-of-concept model were community pharmacists provide PGx testing and clinical decision support	12 months
Ferreri et al. (2014)	USA	18 patients, 2 pharmacists, 1 community pharmacy	Prospective observational study	Determine the feasibility of implementing a PGx service in a community pharmacy	12 months
Haga et al. (2021a)	USA	150 patients, 23 pharmacists, 15 community pharmacies	Prospective cluster interventional randomized study	Explore the feasibility of delivering PGx testing, as a standalone or through an MTM session, through community pharmacies	NA
Haga et al. (2021b)	USA	36 pharmacists, 22 community pharmacies	Prospective cluster interventional randomized study	Assess the feasibility of pharmacist-delivered PGx testing in an independent community pharmacy setting	5 months
Jeiziner et al. (2023)	Switzerland	109 patients, 1 pharmacist, 21 physicians, 1 community pharmacy	Prospective non-randomized observational study	Evaluate, from a patient perspective, the pharmacist-led service of PGx testing and advice (PGx service)	21 months
Levens et al. (2023)	Netherlands	144 patients, 14 pharmacists, 8 physicians, 27 community pharmacies	Prospective, non-randomized, interventional, proof of concept study	Evaluate the feasibility of CYP2C19-guided de-escalation from prasugrel or ticagrelor to clopidogrel in an outpatient setting, facilitated by community pharmacists using a point-of-care (POC) CYP2C19 testing device	4 months
Luke et al. (2021)	Canada	10 pharmacists	Prospective observational qualitative implementation study	Elucidate the factors influencing the integration of PGx testing by pharmacists in their practices and to use the BCW approach to inform future intervention options to support pharmacists with this integration	3 months
Moaddeb et al. (2015)	USA	69 patients, 5 community pharmacies	Prospective observational cross-sectional study	Characterize the experiences and feasibility of offering PGx testing in a community pharmacy setting	4 months
Papastergiou et al. (2017)	Canada	100 patients, 2 community pharmacies	Open-label nonrandomized prospective observational study	Evaluate the feasibility of implementing personalized medication services into community pharmacy practice Assess the number of drug therapy problems identified because of PGx screening	NA
Papastergiou et al. (2021)	Canada	213 patients, 208 physicians, 3 community pharmacies	Prospective interventional randomized controlled trial	Evaluate the impact of PGx guided versus standard antidepressant treatment of depression and anxiety	20 months
Rodríguez-Arcas et al. (2013)	Spain	37 patients, 2 pharmacists, 1 community pharmacy	Prospective interventional cohort study	Assess the feasibility of implementing PGx and PFU* in community pharmacies, and evaluate their potential to enhance treatment effectiveness, improve safety, and optimize drug selection	11 months
Swen et al. (2012)	Netherlands	54 patients, 1 community pharmacy	Prospective observational nonrandomized study	Investigate the feasibility of pharmacy-initiated PGx screening in primary care	24 months
Thornley et al. (2021)	Netherlands	611 patients, 22 community pharmacies	Prospective observational study	Describe how best to implement PGx services within community pharmacy, considering potential barriers and enablers to service delivery and how to address them	12 months
Van der Wouden et al. (2019)	Netherlands	200 patients	Prospective observational cohort study with cross-sectional follow-up	Quantify the feasibility and the real-world impact of implementation of PGx panel in a decision support system	30 months
Van der Wouden et al. (2020)	Netherlands	19 patients, 15 pharmacists	Prospective observational qualitative implementation study	Evaluate study pharmacists’ perceived enablers and barriers for PGx panel-testing among pharmacists participating in a PGx implementation study	NA

PGx*: Pharmacogenetics; MTM*: medication therapy management; PFU*: pharmacotherapeutic follow-up; USA: united states of america, UK: united kingdom.

### 3.3 Study outcomes

The presentation of the themes and sub-themes assessed in the 17 articles is shown in Table 2, with detailed findings of each theme provided in Tables 3–6.

**TABLE 2:** Implementation outcomes used by article.

Implementation variables	Breaux et al. (2020)	Bright et al. (2015)	Papastergiou et al. (2017)	Swen et al. (2012)	Bank et al. (2019)	Ferreri et al. (2014)	Thornley et al. (2021)	Papastergiou et al., 2021	Haga et al. (2021a)	Van der Wouden et al. (2019)	Van der Wouden et al. (2020)	Luke et al. (2021)	Moaddeb et al. (2015)	Rodríguez-Arcas et al. (2013)	Haga et al. (2021b)	Jeiziner et al. (2023)	Levens et al. (2023)
Feasibility																	
Time required for pharmacists to provide PGx services	X		X	X	X	X	X	X		X	X		X	X	X		X
Quality of DNA sampling using saliva kits	X	X	X	X					X					X			X
Evaluation of pharmacists’ interpretation of PGx* test results													X				
Stakeholders’ views on the practical implementation of PGx testing	X			X							X	X	X		X	X	X
Pharmacists’ assessment of patients’ comprehension of PGx testing													X			X	
Appropriateness																	
PGx-related outcomes identified through PGx testing		X	X		X	X	X		X					X			
Adoption																	
Participation rate of patients				X	X	X							X				
Adoption and communication of PGx testing by patients							X		X							X	
Recommendation of PGx testing by pharmacists					X		X						X		X		
Adoption of pharmacist-provided PGx recommendations by physicians		X	X		X	X	X	X						X		X	X
PGx results recorded in the patient’s EMR*							X			X							
Acceptability																	
Perceptions of patients, pharmacists and physicians after undergoing/performing PGx testing	X						X		X		X	X	X		X	X	X

EMR*: electronic medical record; PGx*: Pharmacogenetics.

**TABLE 3:** Findings on the feasibility of PGx testing in community pharmacies by sub-themes from the included articles.

Outcomes	Detailed results
Time required for pharmacists to provide PGx services	
Type of genotyping platform	Microarray-based genotyping [4,5,10,11]
	Multiplex PCR + MALDI-TOF mass spectrometry [1,3,8]
	Real-time PCR (qPCR) [5,7,14]
	Targeted next-generation sequencing (NGS) [1]
	Point-of-care (PoC) PCR testing [17]
To explain PGx testing process to patients	1–10 min [6, 13]
To collect saliva sample	2 min [1]
To perform genotyping	1 day (preliminary, *1 and *2 alleles)/3–5 business days (confirmatory, including LoF* alleles and *17) [2]
	24 h [13]
	1 h [17]
To obtain PGx results	Single-sample processing*: 3–14 days [2,6,7]
	Batch processing*: 6–9 months [1]
PoC testing*	75 min, including pre-test counselling, sample collection, analysis, and 1 h of device runtime [17]
To communicate genetic results to the patients	18 min ± 3–90 min [11,13]
Total patient’s participation time	76.6 (mean) ± 46–120 (range) min [6]
To communicate genetic results to physicians	1–5 min [13]
To obtain prescriber approval	12.6 days ± 1–30 days [6]
Patients’ enrolment completion	23.4 ± 8.3 days [5], 30.1 (mean) ± 14–56 (range) days [6]
Number of tasks performed by pharmacists before and after PGx testing	4.9 ± 1–11 tasks before testing [15]
	2.4 ± 0–6 tasks after testing [15]
Quality of DNA sampling using saliva kits	
Saliva failure rate	1%–16.7% (3% [2], 1% [3], 16.7% [4], 6.7% [9], 5.4% [14], 1.4% [17])
Evaluation of pharmacists’ interpretation of PGx test results	
Correct interpretation of PGx tests by pharmacists	89% [13]
Stakeholders’ views on the practical implementation of PGx testing	
Patients’ view	Difficulties in travelling to the pharmacy (23%) [13]
	Lack of time to participate (15%) [13]
	Willingness to pay for PGx services (62%) [16]
	Cost (10%) [13]
Pharmacists’ view	Infrastructure inefficiencies [11,12,17]
	Staffing challenges [12,17]
	Difficulties in workflow integration [11,12]
	Unclear procedures (e-g time management, lack of standardisation) [1,11,17]
	Sample collection challenges: low DNA yield, dry mouth, and degraded samples requiring re-extraction or additional quality control [1,4]
	Delays in genetic reports reception [1]
	Extended timelines for delivering results [1]
	Lack of reimbursement for PGx testing [11,15,17]
	Lack of reimbursement for associated consultations (e.g., interpretation of PGx tests) [11,15]
	Costs [17]
Pharmacists’ assessment of patients’ comprehension of PGx testing	
Before testing	55% had no concerns or questions about the testing process, the interpretation of results, or its potential impact on their treatment, others inquired about potential medication changes or test costs [13]
After-testing	30%–54% understood the results “very well” [13]
	2%–4% having limited understanding [13]
	Minimal concerns post-results (73% having no further questions, some sought further clarification from their physicians) [13]

[1], Breaux et al. (2020); [2], Bright et al. (2015); [3], Papastergiou et al. (2017); [4], Swen et al. (2012); [5], Bank et al. (2019); [6], Ferreri et al. (2014); [7], Thornley et al. (2021); [8], Papastergiou et al. (2021); [9], Haga et al. (2021a); [10], Van der Wouden et al. (2019); [11], Van der Wouden et al. (2020); [12], Luke et al. (2021); [13], Moaddeb et al. (2015); [14]: Rodríguez-Arcas et al. (2013); [15], Haga et al. (2021b); [16]: Jeiziner et al. (2023); [17], Levens et al. (2023); PGx*, Pharmacogenetics; Time*, time expressed in minutes, days, or months, %*, Rates reported in the studies for each sub-theme; HCPs*, Healthcare Professionals; PoC testing*: Point of Care (PoC) testing refers to genotyping performed directly in the community pharmacy, without sending samples to an external laboratory. Depending on the device, analysis may rely on simplified PCR-based methods or alternative rapid genotyping technologies, providing results within minutes to hours; LoF*: loss of function alleles; Single-sample processing* refers to the immediate PGx, analysis, with results returned on a per-sample basis. Batch processing” refers to the analysis of multiple samples in a batch, delayed from the PGx, sampling, with results returned collectively.

**TABLE 4:** Findings on the appropriateness of PGx testing in community pharmacies by sub-themes from the included articles.

Outcomes	%*
PGx-related outcomes identified through PGx testing	
Rate of medication-related issues*	68% [3]
Patients receiving PGx-based therapeutic recommendations	27% [2], 31% [5], 31.8% [7], 38.9% [6]

[2], Bright et al. (2015); [3], Papastergiou et al. (2017); [5], Bank et al. (2019); [6], Ferreri et al. (2014); [7], Thornley et al. (2021); %*, Rates reported in the studies for each sub-theme.

**TABLE 5:** Findings on the adoption of PGx testing in community pharmacies by sub-themes from the included articles.

Outcomes	%*
Participation rate of patients	
Participation rate	43.9% [6]
Acceptance rate of testing among patients	44.8% [5], 58.1 % [4], 81% [13]
Overall adoption rate	18% [5]
Adoption and communication of PGx testing by patients	
Rate of patients actively requesting for the test	52.3% [7]
Rate of patients sharing PGx results with healthcare professionals	86% of patients consulted their referring physician, and 69% informed other physicians [16], 68.4% of participants shared their PGx results with a doctor, spouse, family member, or another person [9]
Recommendation of PGx testing by pharmacists	
Rate of pharmacists who proposed the test to patients	24.3% [7], 41.5% [5], 100% [13,15]
Adoption of pharmacist-provided PGx recommendations by physicians	
PGx recommendation adopted by physicians	Genetic-related recommendations: 20% [17], 27% [2]; 63.2% [3], 69% [16], 75.2% [8], 82.4% [7], 88.7% [5], 88.9% [6], 100% [14]
	Patients’ statement about physician’s reactions to PGx recommendations [16]: 38% positive
	• 17% neutral
	• 10% negative
	• 36% no statement (due to lack of time or consultation not yet taken place) [17] o Cardiologists refused due to strict adherence to local guidelines (35%)
	o No response from cardiologists (16%)
	o Perceived different therapy indications (11%)
	o Hospital guidelines not aligned (5%)
	o Unknown reasons (7%)
PGx results recorded in the patient’s EMR*	
Rate of PGx result documentation in EMR*	By pharmacists 15.0 % (EHR*) [7]; 96.0% [10] By physicians 67.8% [10]

[2], Bright et al. (2015); [3], Papastergiou et al. (2017); [4], Swen et al. (2012); [5], Bank et al. (2019); [6], Ferreri et al. (2014); [7], Thornley et al. (2021); [8], Papastergiou et al. (2021); [9], Haga et al. (2021a); [10], Van der Wouden et al. (2019); [13], Moaddeb et al. (2015); [14], Rodríguez-Arcas et al. (2013); [15], Haga et al. (2021b); [16], Jeiziner C et al. (2023); [17], Levens et al. (2023); EMR*, electronic medical record; EHR*, electronic health record; PGx*, Pharmacogenetics; %*, Rates reported in the studies for each sub-theme; Documents*, Refer to the lists of concerned substances/recommendations.

**TABLE 6:** Findings on the acceptability of PGx testing in community pharmacies by sub-themes from the included articles.

Outcomes	Stakeholders’ acceptability (%*)
Perceptions of patients, pharmacists and physicians after undergoing/performing PGx testing	
Patients’ perceptions	Satisfaction with the study process (98.2%–100%) [1]
	Satisfaction with PGx testing (97%) [9]
	Patients interested in (pre-emptive) PGx testing (53%) [17]
	Patients recognizing the benefits of PGx testing for their medications (52.6%) [9], (81%) [17]
	Willingness to share PGx results with HCPs (97% [17]), pharmacists (97% [17])
	Pharmacists’ positive role (treatment optimisation, patient consultation, and follow-up, interprofessional communication, patient education and awareness) [9]
	Preference for PGx testing in a pharmacy over hospitals or laboratories (61% [17])
	Perception of the community pharmacy as a suitable location for PGx testing (92%) [17]
	Trust in pharmacists’ ability to perform and interpret PGx tests (92%) [17]
Pharmacists’ perceptions	Overall positive perception of PGx services (85%–100% [1], 86% [17])
	Motivations for offering PGx testing [12]
	• Treatment personalisation (22.7%) [7]
	• Professional development (18.2%) [7]
	• Personal interest (13.6%) [7]
	• Improving compliance with drug regimen (87.5%) [15]
	• Offering the service had helped them improve the relationship they have with other healthcare providers (54.5%) [7]
	Confidence in their ability
	• To make recommendations to physicians (93.8%) [15]
	• To provide counselling to patients (93.8%) [15]
	• To have sufficient time to make recommendations to physicians (87.5%) [15]
	• To be qualified to provide PGx-testing (100%) [15]
	Positive view of their leadership and the future of PGx in pharmacy [11,12]
	Knowledges after training sufficient but decline over time [12] (86.4%) [7]
	Lack of knowledge and training among pharmacists (6.43%) [17]
	Positive feedback from doctors (86.7%) [7]
	Low prescriber engagement [15], (6.43%) [17] (lack of prescribers’ response or interest, communication challenges)
	Lack of physician’s knowledge and awareness about PGx [11,15]
	Lack of timely and effective communication between HCPs [11,15,17]
Physicians’ perceptions	Need for support to put PGx testing into practice (38%) [17]
	Insufficient knowledge of PGx [17]
	Time constraints related to the application of PGx in practice [17]
	Uncertainty about whether PGx results will influence prescribing policies [17]
	Increased administrative burden if results are not automatically displayed in patients’ electronic records [17]
	Finding PGx testing beneficial (88%) [17]

[1], Breaux et al. (2020); [3], Papastergiou et al. (2017); [5], Bank et al. (2019); [7], Thornley et al. (2021); [9], Haga et al. (2021a); [11], Van der Wouden et al. (2020); [12], Luke et al. (2021); [13], Moaddeb et al. (2015); [15], Haga et al. (2021b); [16], Jeiziner et al. (2023); [17], Levens et al. (2023); %*, Rates reported in the studies for each sub-theme, PGx, Pharmacoegenetics; HCPs, Helthcare Professionals.

#### 3.3.1 Feasibility

The feasibility of implementing PGx testing in community pharmacies was explored through five key sub-themes: i) time-related factors, which assess the duration needed for various stages of the testing process; ii) the quality of saliva samples collected for testing; iii) the ability of pharmacists to interpret PGx test results; (iv) the capacity of patients to understand their PGx results and (v) the workflow-related barriers.

In terms of time-related factors associated with PGx testing, 10 studies quantified the duration of specific actions throughout the procedure: explaining the testing procedure to patients ([1–10] minutes) (Ferreri et al., 2014; Moaddeb et al., 2015), collecting saliva samples (2 min) (Breaux et al., 2020), communicating results to physicians ([1–5] minutes) (Moaddeb et al., 2015), communicating results to patients (mean: 18 min; range: [3–90] minutes) (Moaddeb et al., 2015; Van Der Wouden et al., 2020), and obtaining prescribers’ approval (mean: 13 days; range: [1–30] days) (Ferreri et al., 2014). Based on these data, the realistic duration of the entire PGx service per patient is approximately 7 days, when all steps are efficiently processed and when the prescriber approval is fast, to 5–6 weeks, when delays occur in result reporting, as a consequence of poor professional coordination, and lack of prescribers’ response. In one study, pharmacists expressed that delays in receiving PGx results could be too long, potentially hindering timely clinical decision-making (Breaux et al., 2020).

PGx testing at the point of care (PoC) with genetic analyses conducted directly by community pharmacists was performed in a single study (Levens et al., 2023). All other studies (n = 16) relied on external laboratories for the analysis, where trained laboratory personnel performed the genotyping after samples were shipped to the respective facilities. Across the included studies, microarray-based genotyping was used in four studies (Bank et al., 2019; Swen et al., 2012; Van Der Wouden et al., 2019; Van Der Wouden et al., 2020), multiplex PCR combined with MALDI-TOF mass spectrometry in three studies (Breaux et al., 2020; Papastergiou et al., 2017; Papastergiou et al., 2021), real-time PCR (qPCR) in three studies (Bank et al., 2019; Rodríguez-Arcas et al., 2013; Thornley et al., 2021), PoC PCR devices in one study (Levens et al., 2023), and targeted next-generation sequencing (NGS) in one study (Breaux et al., 2020), while seven studies did not specify the genotyping platform used (Bright et al., 2015; Ferreri et al., 2014; Haga et al., 2021a; Moaddeb et al., 2015; Luke et al., 2021; Haga et al., 2021b; Jeiziner et al., 2023).

Regarding the duration of the genetic testing process itself, only three studies explicitly reported this information: one indicated a 1-day turnaround for preliminary testing limited to limited variant alleles, whereas confirmatory clinical testing including additional alleles required three to five business days (Bright et al., 2015); another reported that results were available within 24 h of sample receipt (Moaddeb et al., 2015); and the study employing PoC testing indicated a rapid turnaround, with genetic analysis completed in approximately 1 h (Levens et al., 2023).

Concerning the quality of saliva samples collected for testing, the technical feasibility of the intervention was high, with an overall low failure rate (ranging from 1% to 17%) (Bright et al., 2015; Haga et al., 2021a; Levens et al., 2023; Papastergiou et al., 2017; Rodríguez-Arcas et al., 2013; Swen et al., 2012).

Pharmacists’ ability to interpret PGx test results appears to be sufficient, as evidenced by the study by Moaddeb et al. (Moaddeb et al., 2015), in which 89% of pharmacists correctly interpreted PGx results. The same study also suggests that most patients had a good understanding of PGx testing. Indeed, 55% of patients expressed no concerns or questions regarding the process, the interpretation of results, or the potential impact on their treatment when a brief pre-test discussion (typically lasting 1–5 min) was conducted to explain the purpose of the test. They also mentioned that 30%–54% of patients understood their results “very well”, and 73% had no further questions after the test.

In terms of workflow-related barriers impacting the practical feasibility of the intervention, several studies have highlighted infrastructural challenges that may limit the achievement of PGx testing in the community pharmacies. These include infrastructure inefficiencies (Levens et al., 2023; Luke et al., 2021; Van Der Wouden et al., 2020), staffing challenges (Levens et al., 2023; Luke et al., 2021), difficulties in workflow integration (Luke et al., 2021; Van Der Wouden et al., 2020), and unclear procedures (e.g., time management, lack of standardization) (Breaux et al., 2020; Levens et al., 2023; Van Der Wouden et al., 2020) notably in relation to the collection and the process of saliva sampling (Breaux et al., 2020; Swen et al., 2012). Moreover, difficulties in travelling to the pharmacy and limited time availability to perform the test was expressed by patients (Moaddeb et al., 2015). In addition, financial constraints such as the cost of the PGx tests themselves, the associated cost of test interpretation and patient follow-up, and the lack of reimbursement mechanisms were also commonly reported by pharmacists and patients (Levens et al., 2023; Moaddeb et al., 2015; Haga et al., 2021b; Van Der Wouden et al., 2020). Nevertheless, 62% of patient expressed their willingness to pay for such tests (Jeiziner et al., 2023).

#### 3.3.2 Appropriateness

The appropriateness of PGx testing in community pharmacies was assessed by (i) evaluating the number of therapeutic recommendations formulated based on PGx test results and (ii) identifying medication-related issues, such as risks of toxicity, reduced effectiveness, or altered metabolism, arising from genetic variability.

Regarding (i), several studies reported that PGx-informed therapeutic recommendations were formulated for 27%–39% of patients, depending on the study (Bank et al., 2019; Bright et al., 2015; Ferreri et al., 2014; Papastergiou et al., 2017; Rodríguez-Arcas et al., 2013; Thornley et al., 2021). Concerning (ii), one study by Papastergiou et al. (Papastergiou et al., 2017) found that 68% of the identified medication-related issues were directly related to PGx test results. These issues predominantly involved antidepressants (25%), statins (19.1%) and clopidogrel (17.6%), and included interventions such as changes in therapy, dosage adjustments, drug discontinuation, or increased monitoring.

#### 3.3.3 Adoption

The sub-theme of adoption of PGx testing was explored through four key aspects: (i) participation rates of eligible patients, (ii) adoption and communication of PGx testing by patients, (iii) recommendation of PGx testing by pharmacists, (iv) adoption rate of pharmacist-provided PGx recommendations by prescribing physicians, and (iv) documentation of PGx information in electronic medical records (EMRs) by healthcare professionals.

Patient participation rates encompass several dimensions. First, they include the proportion of eligible patients who agreed to participate in the study, reported in a single study at 43.9% (Ferreri et al., 2014). They also cover the proportion of patients who accepted testing among those to whom pharmacists proposed it, with reported rates ranging from 45% to 81% (Bank et al., 2019; Moaddeb et al., 2015; Swen et al., 2012). Finally, they include the overall adoption rate, defined as the proportion of all eligible patients who ultimately underwent PGx testing, which was reported in one study at 18% (Bank et al., 2019). Adoption and communication of PGx testing by patients encompassed two main aspects. First, one study reported that 52.3% of patients actively requested PGx testing (Thornley et al., 2021). Second, two studies showed that 69% of patients shared their PGx results on their own initiative, both with physicians involved in their care (Haga et al., 2021a; Jeiziner et al., 2023) and with their relatives (Haga et al., 2021a). Recommendation of PGx testing by pharmacists also varied considerably across studies. In fact, reported rates of pharmacists who proposed testing to patients ranged widely, from 24.3% (Thornley et al., 2021) to 41.5% (Bank et al., 2019), while in some studies all included patients were offered testing by their pharmacist (Moaddeb et al., 2015; Haga et al., 2021b).

Physician adoption of pharmacist-provided PGx recommendations varied considerably across studies, ranging from 27% to 100% (Bank et al., 2019; Bright et al., 2015; Ferreri et al., 2014; Papastergiou et al., 2017; Papastergiou et al., 2021; Rodríguez-Arcas et al., 2013; Jeiziner et al., 2023; Thornley et al., 2021). However, one study focusing specifically on cardiologists reported a notably lower adoption rate of 20% (Levens et al., 2023). This study identified several barriers to the uptake of PGx recommendations, including strict adherence to alternative local guidelines (35%), inconsistencies with hospital policies (5%), and differing views on the appropriate clinical use of PGx testing (11%). The overall integration of PGx results into EMRs varied considerably across studies. One study reported relatively high documentation rates, with 96% of pharmacists and 68% of physicians recording PGx results in patients’ EMRs (Van Der Wouden et al., 2019). In contrast, another study found significantly lower documentation rates among pharmacists, with only 15% entering PGx data into the electronic health record (EHR), thereby limiting accessibility for other prescribers (Thornley et al., 2021).

#### 3.3.4 Acceptability

The sub-theme of acceptability in PGx testing was examined through three main aspects: (i) patients’ interest and satisfaction, (ii) pharmacists’ engagement, confidence, and perceptions in delivering PGx services, (iii) and physicians’ perceptions in incorporating PGx information into clinical practice.

Overall, patients demonstrated a strong interest and satisfaction with PGx testing, supporting its acceptability and perceived value in community pharmacy settings: 53% of patients expressed interest in preemptive PGx testing (Levens et al., 2023); 98.2%–100% of patients expressed satisfaction across various aspects of the process (including the information and explanations received, understanding of the study’s purpose, opportunity for discussion, comfort with participation, and support for the role of community pharmacies in delivering genetic testing) (Breaux et al., 2020); 81% of patients acknowledged its usefulness (Haga et al., 2021a; Levens et al., 2023); 92% of patients show confidence in the pharmacists’ competence to perform and interpret PGx tests (Levens et al., 2023). Additionally, community pharmacies were perceived as an appropriate location for PGx testing by most patients, with 61% preferring this setting over hospitals or laboratories (Levens et al., 2023).

Pharmacists generally showed strong acceptance and engagement with PGx testing, not only in performing the tests but also in supporting their integration into practice. This engagement was reflected through several key findings. First, the overall positive perception of the PGx service among pharmacists ranged from 85% to 100% (Breaux et al., 2020; Levens et al., 2023; Luke et al., 2021; Haga et al., 2021b; Thornley et al., 2021). Second, their motivation was primarily driven by the desire to improve patient adherence to treatment regimens, as reported by 87.5% of participants (Haga et al., 2021b). Third, they expressed high levels of confidence in their ability to make recommendations to physicians (93.8%) (Haga et al., 2021b) and to counsel patients on their PGx test results (93.8%) (Haga et al., 2021b), and by their perception of leadership in this innovative field (Luke et al., 2021; Van Der Wouden et al., 2020). Finally, after receiving specific training, 86% of pharmacists considered their knowledge sufficient to use PGx in practice, though this perception tended to decline over time (Luke et al., 2021; Thornley et al., 2021). However, pharmacist’s perception of interprofessional collaboration remained inconsistent. While one study reported a high rate of positive feedback from physicians (86.7%) (Thornley et al., 2021), other studies highlighted pharmacists’ perceptions of limited physician engagement, low responsiveness, and persistent communication challenges (Levens et al., 2023; Haga et al., 2021b).

Regarding physicians’ perceptions of incorporating PGx information into clinical practice, a substantial majority (88%) acknowledged that PGx testing may offer clinical benefits by supporting personalized treatment decisions (Levens et al., 2023). In the same study, 38% of physicians expressed a need for support to integrate PGx into their practice, and others citied insufficient knowledge, time constraints, and uncertainty about the impact of PGx results on prescribing decisions.

## 4 Discussion

To the best of our knowledge, this study is the first to comprehensively evaluate the implementation of PGx testing in the routine setting of community pharmacies, with a specific focus on four key implementation outcomes: feasibility, appropriateness, adoption and acceptability.

Following the implementation of PGx testing, patients, pharmacists, and physicians provided insights into their perceived usefulness, practical feasibility, and integration into community pharmacy workflows. From the patient perspective, acceptance and satisfaction with PGx testing in community pharmacies were consistently high. Patients were strongly confident in pharmacists’ competence to perform and interpret the tests. PGx testing was regarded as useful and clinically relevant, with community pharmacies widely seen as an appropriate and convenient setting to perform the test. Nevertheless, practical constraints, such as difficulties travelling to the pharmacy and limited availability to complete testing, were noted as potential barriers.

While participation and acceptance rates varied considerably across studies, the observation that approximately half of patients proactively requested PGx testing underscores a strong patient-driven demand for PGx testing. This level of initiative is notable, as it suggests that patients are not only receptive to PGx when offered, but also actively engaged in seeking it, reflecting increasing awareness and perceived value of personalized medicine. At the same time, the variability in pharmacist-initiated recommendations indicates that patient access still largely depends on professional gatekeeping, and the overall adoption rate among all eligible patients remains modest. Moreover, the fact that a substantial proportion of patients voluntarily shared their results with healthcare providers and relatives demonstrates that patients view PGx as relevant beyond the immediate prescribing context. Altogether, these patterns point to a growing patient-driven dynamic for PGx implementation, which could represent an important lever to complement professional initiatives but also signal the need to better align healthcare system structures.

However, translating this enthusiasm into routine practice also depends on the readiness of pharmacists, who, while highly motivated, face a distinct set of challenges. Indeed, pharmacists demonstrated strong acceptance and active engagement with PGx testing. Their motivation was frequently driven by the desire to improve patient adherence and to play a proactive role in optimizing treatment outcomes. They expressed confidence in counselling patients and making clinical recommendations to physicians, and many viewed themselves as leaders in advancing this innovative service. Targeted training further enhanced their confidence and preparedness to implement PGx in practice, although in some cases this sense of readiness declined over time. Despite these positive perceptions, several challenges were noticed. Delays in receiving results, particularly when reliant on external laboratories, were seen as obstacles to timely clinical decision-making. Integration into existing workflows was further complicated by infrastructure constraints, staffing shortages, and unclear or non-standardized procedures. Financial considerations, including the high cost of tests, interpretation, and follow-up in the absence of reimbursement, were also reported as barriers. Interprofessional collaboration between pharmacists and physicians was variable: while some reported constructive and supportive engagement, others described limited responsiveness and ongoing communication challenges, underscoring the need for more effective and standardized collaboration pathways. Compared to pharmacists, physicians’ perceptions were favorable but more cautious. They acknowledged the potential of PGx testing to enhance personalized prescribing but highlighted barriers such as insufficient knowledge, time constraints, and uncertainty about how to integrate results into practice.

One recurrent theme underlying several of these challenges is the insufficient PGx training among healthcare professionals, particularly physicians. Our review found no evidence of PGx-specific education targeting physicians in the included studies, which may partially explain their inconsistent engagement with PGx recommendations. This is consistent with findings from a large US study, where despite strong theoretical support for PGx (97.6% agreement), only 10.3% of physicians felt adequately informed, and less than 30% had received relevant training (Stanek et al., 2012). These figures highlight a clear gap between perceived importance and actual preparedness. The need for tailored educational strategies is further supported by studies showing a correlation between prescribers’ training and their likelihood of accepting PGx-based recommendations (Crews et al., 2011; Cicali et al., 2019; Bain et al., 2018). While this issue affects various healthcare professionals, the contrast with pharmacists is notable: most received targeted PGx education, which appears to enhance their confidence and ability to implement PGx services. However, even among pharmacists, insufficient training can undermine feasibility by increasing the risk of misinterpretation, weakening communication, and limiting the adoption of recommendations, as shown by Hayashi et al. (Hayashi et al., 2022).

Timeliness is a key determinant of the clinical utility of PGx testing. Although reported timeframes were generally manageable, delays in receiving results remain a major barrier to timely therapeutic decisions (Breaux et al., 2020). PoC testing has emerged as a promising solution, offering rapid results directly within the pharmacy. Its feasibility and reliability have been demonstrated, with one study reporting a 98.6% success rate for *CYP2C19*-guided testing (Levens et al., 2023) and up to 97% concordance with laboratory-based methods (Wirth et al., 2016). However, faster testing must be complemented by effective data integration. Our findings and previous studies highlight persistent inconsistencies in how and to which extent PGx results are documented in EHRs, limiting their accessibility across care settings (Thornley et al., 2021). Integrating PGx data into EHRs, particularly when supported by clinical decision support (CDS) tools, could significantly improve clinical responsiveness, by enabling real-time identification of gene–drug interactions and guiding evidence-based treatment decisions (Van Der Wouden et al., 2019). Such infrastructure would also support pharmacists in interpreting results and adjusting therapies accordingly.

Ineffective communication between healthcare professionals, particularly between pharmacists and physicians, emerged as a recurring barrier to the implementation of PGx testing in community pharmacy (Levens et al., 2023; Haga et al., 2021b; Van Der Wouden et al., 2020), echoing previous findings in the literature (Aref et al., 2003). Such communication gaps may reduce the clinical responsiveness of PGx services and limit physicians’ acceptance of pharmacist-led recommendations. Most studies reported the use of indirect communication methods, such as written reports, electronic messages, or patient-mediated transmission (Levens et al., 2023; Haga et al., 2021b). While these approaches are practical and time-efficient, they often lack interactivity and opportunities for real-time clarification, which may contribute to a lower uptake of PGx recommendations. In contrast, direct communication, via face-to-face or telephone discussions, was less commonly implemented but appears more effective. For instance, Thornley et al. (Thornley et al., 2021) reported an in-person communication model that resulted in a high adoption rate of pharmacist recommendations (82.4%), suggesting that such methods can enhance mutual trust and clinical integration. To address these communication-related challenges, a structured implementation guide has been developed to support pharmacist-led PGx testing and promote more interactive, standardized communication pathways between professionals (Stäuble et al., 2022).

The successful implementation of PGx testing in community pharmacy is closely tied to both economic and policy contexts and is further reinforced by the growing body of clinical evidence supporting its utility.

From an economic standpoint, although test costs are gradually declining, the initial investment, combined with expenses for result interpretation, clinical decision support, and follow-up, remains a barrier to adoption, particularly in the absence of reimbursement mechanisms (Patel et al., 2025). Cost-effectiveness analyses indicate that PGx testing can be economically viable in certain therapeutic areas, such as mental health (Groessl et al., 2018), cardiology (Zhu et al., 2020), or in the context of high-cost treatments such as oncology (Swen et al., 2023), by reducing adverse drug reactions and improving treatment efficiency. However, such evidence remains heterogeneous and context-dependent, with payers often requiring robust local data before committing to systematic funding. Reimbursement policies therefore represent a major driver: the existence of a clear reimbursement mechanism facilitates large-scale adoption, whereas the absence of coverage restricts access either to research settings or to patients able to bear the costs themselves.

Despite the central importance of economic considerations, costs were only sporadically assessed in the included studies, with limited information available. Jeiziner et al. (Jeiziner et al., 2023) reported an estimated €400 for laboratory analysis and €300 for pharmacist counselling, which included two 30-min consultations, sample collection, and at least 40 min for preparing a recommendation letter. Breaux et al. (Breaux et al., 2020) did not specify the PGx testing cost itself, but estimated therapy adjustment costs at approximately CAD 24.15 per patient annually and highlighted that managing non-responders could reach USD 10,000 per patient per year. Similarly, Levens et al. (Levens et al., 2023) estimated a net operational cost of €43 per patient when accounting for savings from therapeutic de-escalation. The remaining 11 studies provided no cost-related data, underlining a major evidence gap regarding the financial dimension of PGx implementation in community pharmacy. This scarcity of cost data significantly limits the ability to evaluate the real-world economic feasibility of PGx testing in these settings.

Moreover, economic viability cannot be fully separated from policy context: reimbursement decisions, legal authorizations, and regulatory structures strongly condition the extent to which PGx services can be deployed. From a policy perspective, legislative frameworks play a decisive role in the adoption of PGx testing. Legal provisions can either facilitate or restrict implementation across jurisdictions. For instance, in certain Canadian provinces, commercial tests are available through several providers [e.g., Pillcheck (Inc, 2025)], enabling pharmacists to offer and interpret PGx tests for patients. In Switzerland, community pharmacists are now legally authorized to prescribe certain PGx tests, following the revision of the Swiss Law on Human Genetic Testing (LAGH) in 2018 (Suisse, 2018) and the adoption of the 2022 Ordinance on Genetic Testing (OAGH) (Suisse, 2022). Yet, reimbursement remains restricted to a limited list of medications, and often requires prescription by specialist physicians, thereby limiting broader access. At a broader level, included studies were conducted in the Netherlands, the USA, Canada, Spain, and Switzerland. These countries have well-structured healthcare systems, with organizational models, reimbursement frameworks, and legal requirements that may facilitate the integration of PGx testing within precision medicine.

Beyond these national contexts, there is a growing tendency to integrate PGx into broader genomic medicine initiatives. In the United Kingdom, for example, the Network of Excellence for Pharmacogenomics and Medicines Optimization was launched in 2024 as part of the NHS Genomic Networks of Excellence program. This initiative aims to coordinate PGx implementation across the health system, particularly in primary care, by fostering partnerships between the NHS, academia, industry, and clinical stakeholders (McDermott et al., 2025). Similarly, in France, the Plan France Médecine Génomique 2025 (Mdl, 2025; PFMG 2025 contributors, 2025) seeks to integrate genomics into routine diagnostic and therapeutic practice by developing a national network of high-throughput sequencing platforms and dedicated bioinformatics tools for the analysis, interpretation, and integration of genomic data into clinical pathways.

Finally, clinical evidence and guidelines further strengthen the case for PGx adoption. Several clinical platforms, such as those provided by the Clinical Pharmacogenetics Implementation Consortium (CPIC) (Relling and Klein, 2011) and the Dutch Pharmacogenetics Working Group (DPWG) (Swen et al., 2008; Swen et al., 2011), deliver clear, evidence-based gene–drug recommendations, facilitating the translation of genetic information into clinical decision-making. In addition, specific guidance documents, such as those addressing clopidogrel therapy in combination with CYP2C19 testing (Lee et al., 2022), or manufacturer-driven requirements such as pre-treatment testing before siponimod initiation (Díaz-Villamarín et al., 2022), illustrate how PGx is progressively embedded into clinical practice standards and regulatory frameworks.

A key strength of our methodology lies in its exclusive focus on patients and healthcare professionals with direct experience of PGx testing, providing a more accurate reflection of real-world practice. In contrast to the recent scoping review by Aref et al. (2003), which applied different implementation frameworks and included studies with and without real-world PGx implementation data, our study, by focusing exclusively on empirical data from pharmacist-led PGx services in community pharmacies up to 2025, enhances the relevance and applicability of the findings. Our approach also limits speculative responses and bias from participants without direct exposure, a limitation frequently reported in previous studies, where lack of practical experience was associated with lower confidence, negative perceptions, and reduced engagement with PGx-related behaviors (Youssef et al., 2022; Virelli et al., 2021).

Yet, our comprehensive search has several limitations that should be acknowledged. First, the quality of some included studies could be questioned, particularly those involving small sample sizes, which may reduce the strength and generalizability of their findings. Secondly, heterogeneity in methodologies and outcomes measured was challenging and introduced subjectivity in the interpretation of the classification. Thirdly, the use of the Proctor framework may have led to the omission of certain organizational factors influencing the implementation of PGx in community pharmacy. Nevertheless, it remains a widely used tool in implementation science to assess implementation success. Fourthly, the lack of detailed economic data may limit the ability to thoroughly assess the financial impact of PGx implementation in community pharmacy. Although some studies acknowledged financial barriers to PGx implementation (Levens et al., 2023; Haga et al., 2021b; Van Der Wouden et al., 2020), most did not report detailed cost data for patients or healthcare systems. Only two studies (Breaux et al., 2020; Ferreri et al., 2014) provided partial economic insights, insufficient for a comprehensive analysis. This highlights the need for longitudinal studies incorporating both direct and indirect costs to better evaluate the economic impact of PGx services in community pharmacy. Fifthly, most studies relied on quantitative methods and focused primarily on pharmacists, with limited attention to the views of physicians and patients. Future research should adopt a mixed-methods approach, combining quantitative surveys with in-depth qualitative interviews, to capture the contextual, emotional, and relational dimensions of PGx integration in clinical practice. Finally, we did not conduct a formal critical appraisal of study quality. While the robustness of individual studies could not be formally assessed, we have clearly reported the characteristics and context of each included study to allow readers to interpret the findings with appropriate caution.

This scoping review highlights several actionable strategies to advance the integration of PGx testing in community pharmacies. Evidence shows that direct pharmacist–physician contact, such as face-to-face or telephone communication, increases uptake of PGx recommendations. Structured, interactive communication protocols, for example, the pharmacist-led PGx implementation guide by Stäuble et al. (2022), can help achieve this. The guide outlines a six-step process encompassing patient referral, pre-test counselling, PGx testing, structured medication review, communication of results to both patient and prescriber, and follow-up to assess implementation. This standardized workflow clarifies roles, facilitates timely bidirectional communication, and supports consistent adoption of PGx-guided interventions.

PoC testing models achieving rapid (<1 h) turnaround with high concordance to laboratory testing could be incorporated into pharmacy workflows with clearly defined counselling, analysis, and reporting steps. Best practice reporting involves promptly documenting raw results, translating them into clinical phenotypes with guideline-based recommendations, securely sharing the summary with prescribers and patients, and archiving it for future use. This ensures rapid results are consistently interpreted, actionable, and integrated into ongoing treatment decisions.

From a policy perspective, examples from Switzerland, Canada, and the UK demonstrate how enabling legislation and targeted funding can accelerate PGx integration. One promising approach is the implementation of pilot reimbursement models in which PGx services are temporarily funded for a defined population or period to generate real-world evidence. Such pilots should evaluate both clinical impact—such as reduced adverse drug reactions or improved therapeutic outcomes—and economic value, including potential cost savings. The resulting data can then provide a robust foundation for policymakers and payers to decide on permanent, large-scale reimbursement.

The economic case for PGx testing in community pharmacies is promising but remains heterogeneous. While studies suggest potential cost savings through reduced adverse drug reactions, fewer hospitalizations, and optimized prescribing, evidence is inconsistent across therapeutic areas and health systems. Reimbursement gaps continue to limit access, often confining services to research contexts or to patients able to self-fund. Given that healthcare costs, prescribing patterns, and pharmacy service structures vary widely by country and region, local health-economic evaluations are essential to capture the true value of PGx in each setting. These should measure not only direct financial impacts but also indirect benefits such as improved adherence, prevention of treatment failures, and enhanced patient satisfaction. Generating such evidence can guide the development of sustainable reimbursement models and supportive legislation, enabling the large-scale, equitable integration of PGx testing into community pharmacy practice.

## 5 Conclusion

Our findings advocate for the integration of PGx testing into community pharmacies to support personalized and tailored patient care. By enabling timely access to actionable genetic information, PGx testing facilitates optimized medication management. Future efforts should focus on developing PGx training programs for healthcare professionals, incorporating relevant PGx data systematically into EMRs and EHRs, and fostering innovative effective collaboration between pharmacists and prescribing physicians to ensure broader adoption and successful implementation of PGx services.

## Data Availability

The original contributions presented in the study are included in the article/Supplementary Material, further inquiries can be directed to the corresponding author.
