# Supplementary material for: Implementing pharmacogenetic testing in community pharmacy practice: a scoping review
Source: Front Pharmacol. 2025 Sep 22;16:1659875. doi: 10.3389/fphar.2025.1659875 (PMC12497854; doi:10.3389/fphar.2025.1659875)
Supplement: Supplementary file 1 [file Supplementaryfile1.docx]

Supplementary materials:

Appendix 1: Peer-review strategies according to the Peer Review of Electronic Search Strategies (PRESS) checklist

| Database | Research equation |
| --- | --- |
| Pubmed | ("Community Pharmacy Services"[Mesh] OR "chemist shop*"[tiab] OR "chemist's shop*"[tiab] OR apothecar*[tiab] OR (("Pharmacies"[Mesh] OR "Pharmacy"[Mesh:NoExp] OR "Pharmacists"[Mesh] OR pharmacy[tiab] OR pharmacies[tiab] OR pharmacist*[tiab] OR dispensary[tiab] OR dispensaries[tiab]) AND ("Community Health Services"[Mesh] OR "Primary Health Care"[Mesh:NoExp] OR "Ambulatory Care"[Mesh:NoExp] OR communit*[tiab] OR "primary care"[tiab] OR "Primary health care"[tiab] OR "Primary healthcare"[tiab] OR retail[tiab]))) AND ("Pharmacogenetics"[Mesh] OR "Pharmacogenomic Testing"[Mesh] OR "Genetic Testing"[Mesh:NoExp] OR "Genetic Counseling"[Mesh] OR "Genetic Services"[Mesh:NoExp] OR pharmacogenetic*[tiab] OR pharmacogenomic*[tiab] OR (("genetics"[sh] OR genetic*[tiab] OR genotyp*[tiab] OR genomic[tiab]) AND (test*[tiab] OR detection*[tiab] OR screening*[tiab] OR service*[tiab] OR sequencing[tiab] OR counselling[tiab] OR counseling[tiab] OR ((Buccal[tiab] OR saliva[tiab]) AND (swab[tiab] OR smear[tiab]))))) |
| Embase | ('pharmacy (shop)'/de OR 'community pharmacist'/de OR ((chemist NEAR/3 shop*) OR apothecar*):ab,ti,kw OR (('pharmacist'/de OR (pharmacy OR pharmacies OR pharmacist* OR dispensary OR dispensaries):ab,ti,kw) AND ('primary health care'/exp OR 'ambulatory care'/de OR 'community care'/de OR (communit* OR "primary care" OR "Primary health care" OR "Primary healthcare" OR retail):ab,ti,kw))) AND ('pharmacogenetics'/de OR 'pharmacogenomics'/de OR 'genetic service'/exp OR (pharmacogenetic* OR pharmacogenomic* OR ((genetic* OR genotyp* OR genomic) NEAR/12 (test* OR detection* OR screening* OR service* OR sequencing OR counselling OR counseling)) OR ((genetic* OR genotyp* OR genomic) AND ((Buccal OR saliva) NEAR/3 (swab OR smear)))):ab,ti,kw) NOT (([conference abstract]/lim OR [conference review]/lim) NOT [2019-2030]/py) |
| Cochrane | #1  (((chemist NEAR/3 shop*) OR apothecar*):ab,ti,kw OR (((pharmacy OR pharmacies OR pharmacist* OR dispensary OR dispensaries):ab,ti,kw) AND ((communit* OR "primary care" OR "Primary health care" OR "Primary healthcare" OR retail):ab,ti,kw))) AND ((pharmacogenetic* OR pharmacogenomic* OR ((genetic* OR genotyp* OR genomic) NEAR/12 (test* OR detection* OR screening* OR service* OR sequencing OR counselling OR counseling)) OR ((genetic* OR genotyp* OR genomic) AND ((Buccal OR saliva) NEAR/3 (swab OR smear)))):ab,ti,kw)  #2  ((Trial registry record):pt) OR ((conference proceeding):pt)  #1 NOT #2 |
| Web of Science | TS=((((chemist NEAR/2 shop*) OR apothecar*) OR ((pharmacy OR pharmacies OR pharmacist* OR dispensary OR dispensaries) AND (communit* OR "primary care" OR "Primary health care" OR "Primary healthcare" OR retail))) AND (pharmacogenetic* OR pharmacogenomic* OR ((genetic* OR genotyp* OR genomic) NEAR/11 (test* OR detection* OR screening* OR service* OR sequencing OR counselling OR counseling)) OR ((genetic* OR genotyp* OR genomic) AND ((Buccal OR saliva) NEAR/2 (swab OR smear))))) |

Appendix 2: Taxonomy of implementation themes according to Proctor criteria (Proctor, Silmere et al. 2011)

| Implementation themes | Definitions |
| --- | --- |
| Feasibility | “Feasibility is defined as the extent to which a new treatment, or an innovation, can be successfully used or carried out within a given agency or setting” |
| Appropriateness | “Appropriateness is the perceived fit, relevance, or compatibility of the innovation or evidence-based practice for a given practice setting, provider, or consumer; and/or perceived fit of the innovation to address a particular issue or problem.” |
| Adoption | «Adoption is defined as the intention, initial decision, or action to try or employ an innovation or evidence-based practice” |
| Acceptability | “Acceptability is the perception among implementation stakeholders that a given treatment, service, practice, or innovation is agreeable, palatable, or satisfactory.” |
| Implementation cost | “Implementation cost is defined as the cost impact of an implementation effort.” |
| Penetration | “Penetration is defined as the integration of a practice within a service setting and its subsystems.” |
| Sustainability | “Sustainability is defined as the extent to which a newly implemented treatment is maintained or institutionalized within a service setting’s ongoing, stable operations.” |

References :

Proctor, E., H. Silmere, R. Raghavan, P. Hovmand, G. Aarons, A. Bunger, R. Griffey and M. Hensley (2011). "Outcomes for implementation research: conceptual distinctions, measurement challenges, and research agenda." Adm Policy Ment Health **38**(2): 65-76.
